# Antimicrobial and Immunomodulatory Effects of Punicalagin and Meropenem in a Murine Model of Sublethal Sepsis

**DOI:** 10.3390/antibiotics14070626

**Published:** 2025-06-20

**Authors:** Liliane dos Santos Rodrigues, Priscila Mendonça Mendes, André Alvares Marques Vale, José Lima Pereira-Filho, Elizabeth Soares Fernandes, Joicy Cortez de Sá Sousa, Márcia Cristina Gonçalves Maciel, Valério Monteiro-Neto

**Affiliations:** 1 Graduate Program in Biodiversity and Biotechnology of the Amazon, Federal University of Maranhão, São Luís 65080-805, Brazil; lilik.beq@hotmail.com; 2 Graduate Program in Health Sciences, Federal University of Maranhão, São Luís 65080-805, Brazil; mendes.priscila@discente.ufma.br (P.M.M.); andre_amvale@hotmail.com (A.A.M.V.); jlp.filho@outlook.com (J.L.P.-F.); 3 Department of Animal and Veterinary Sciences, Aarhus University, 8830 Tjele, Denmark; elizabeth.fernandes@anivet.au.dk; 4 Pharmacy Department, Federal University of Maranhão, São Luís 65080-805, Brazil; joicy.sa@ufma.br; 5 Department of Cell Biology, University of Brasilia, Brasília 70910-900, Brazil; marcia.maciel@unb.br; 6 Health Sciences Center, Federal University of Maranhão, São Luís 65080-805, Brazil

**Keywords:** punicalagin, sepsis, antimicrobial activity, immunomodulatory activity

## Abstract

**Background:** Punicalagin (Pg), a major ellagitannin derived from pomegranates, possesses antimicrobial, antioxidant, and immunomodulatory properties, suggesting its potential as an adjunctive therapy for sepsis. **Objectives:** This study investigated the synergistic effects of punicalagin and meropenem in a murine model of sublethal sepsis induced by cecal ligation and puncture (CLP). **Methods:** Mice were treated with punicalagin and meropenem, and multiple parameters were analyzed, including hematological indices, bacterial burden, lymphoid organ cellularity, cytokine profiles (IL-2, IL-4, IL-6, IL-10, IL-17, IFN-γ, TNF-α), nitric oxide (NO) production, and organ histopathology. **Results:** Punicalagin enhanced NO-mediated antimicrobial responses, increased neutrophil migration, preserved lymphoid cellularity, and significantly reduced the bacterial translocation. Combined therapy with meropenem improved systemic IL-10 levels and mitigated histopathological damage in the liver, kidney, intestine, and lung. Importantly, punicalagin did not induce thrombocytopenia. **Conclusions:** These results support the potential of punicalagin as an adjunctive agent to antibiotics for sepsis treatment, offering both antimicrobial and immunoregulatory benefits. Further studies are required to explore its clinical applicability.

## 1. Introduction

Punicalagin, a predominant phenolic compound abundantly found in pomegranate peel, juice, and seeds, is a water-soluble ellagitannin known for its diverse nutritional and medicinal benefits [1,2,3]. This compound exhibits a wide range of biological activities, including anticancer [4,5], antioxidant [6,7], antiviral [8,9], and antibacterial properties [10,11], underscoring its therapeutic potential in various medical applications.

As an antioxidant, punicalagin effectively reduces oxidative stress, as demonstrated by its protective effects in hypertensive pregnant rats [6] and ability to mitigate diabetic liver damage [7]. Its antiviral properties have been demonstrated by its ability to suppress viral replication in infections caused by Herpes Simplex Virus 1 (HSV-1), Hepatitis C Virus (HCV), Respiratory Syncytial Virus (RSV) [9], SARS-CoV-2 via NSP13 helicase inhibition [12], and various influenza strains through neuraminidase inhibition [8].

Punicalagin’s antibacterial effectiveness is notable for its inhibitory effect on biofilm formation by pathogens such as *Staphylococcus aureus* [11]. It also enhances antibiotic efficacy, notably against methicillin-resistant *S. aureus* (MRSA), by downregulating *mecA*, a key mechanism underlying antibiotic resistance [13]. In addition, punicalagin exerts a strong bactericidal effect against *Salmonella typhimurium* by disrupting its cell membrane [10].

Given the broad-spectrum, antimicrobial properties of punicalagin, its potential application in the treatment of sepsis, a condition with a significant global burden, warrants further exploration. According to the World Health Organization [14], sepsis affects approximately 49 million individuals annually, resulting in approximately 11 million deaths. Major contributing factors to this high mortality rate include delayed diagnosis, inadequate treatment regimens, antimicrobial resistance, and limitations in healthcare infrastructure, particularly among neonates and intensive care unit (ICU) patients.

Considering the necessity for effective antimicrobials in sepsis treatment, meropenem, a broad-spectrum carbapenem, plays a crucial role. It is a highly effective antibiotic against both Gram-positive and Gram-negative pathogens, including *Pseudomonas aeruginosa*, *Acinetobacter* spp., and anaerobes [15]. It is also one of the most frequently prescribed antibiotics for the empirical treatment of severe infections [16]. Furthermore, meropenem-based regimens offer superior antibiotic coverage for spontaneous bacterial peritonitis in patients with severe cirrhosis compared to third-generation cephalosporins [17] and for other sepsis resulting from secondary peritonitis [18].

Despite its promising therapeutic properties, the potential of punicalagin as an adjunctive therapy for sepsis, particularly in combination with conventional antibiotics, remains inadequately explored. To address this critical gap, this study aimed to investigate the synergistic effects of punicalagin and meropenem on microbial load reduction and immune response modulation in a murine model of sepsis. This study provides insights into novel therapeutic strategies that could significantly enhance treatment outcomes and patient survival in the management of clinical sepsis.

## 2. Results

### 2.1. Effect of Associate Treatment (Pg ATB) on Nitric Oxide Levels and CFUs

**Figure 1:**
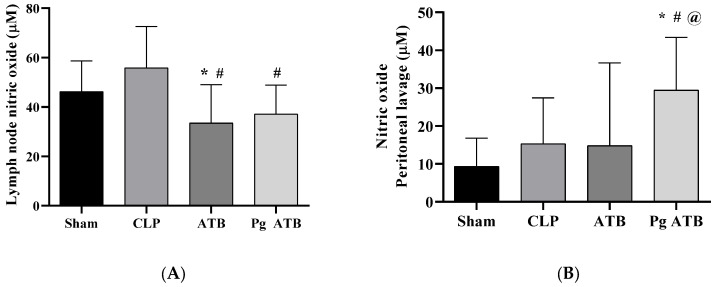
Effects of punicalagin (Pg) and antibiotic (ATB) treatment in septic mice. (**A**) Nitric oxide index in the mesenteric lymph nodes and (**B**) peritoneal lavage. The animals were divided into four groups: Sham, animals that underwent the surgical process but without cecal ligation and without treatment; cecal ligation and puncture (CLP), animals that had sepsis induced by cecal ligation and perforation without treatment; ATB, animals that had sepsis induced by cecal ligation and perforation and were treated with meropenem; and Pg ATB, animals that had sepsis induced by cecal ligation and perforation and were treated with Pg and ATB. ANOVA statistical tests were used, followed by the Tukey post-test, and significant differences are represented by * in relation to the Sham group, # in relation to the CLP, and @ in relation to ATB. Data are presented as mean ± standard deviation (SD), with *p* < 0.05.

Treatment with an antibiotic (ATB) and Pg in septic mice resulted in a significant reduction in nitric oxide (NO) levels in the mesenteric lymph nodes (*p* < 0.001) compared to those in the CLP group (Figure 1A). In addition, it increased NO levels in the peritoneal lavage compared to the other groups (*p* < 0.001) (Figure 1B).

**Figure 2:**
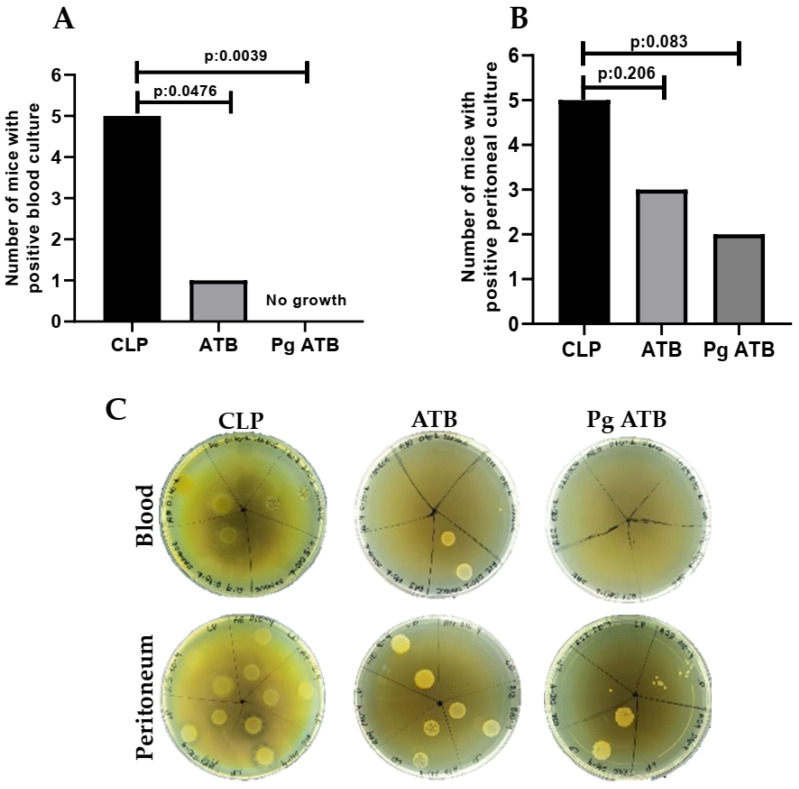
Number of mice with positive culture results. (**A**) Blood cultures: Significant reductions were observed in the ATB and Pg + ATB groups compared to the CLP group. (**B**) Peritoneal cultures: Trends suggested reduced positivity in treated groups, but differences were not statistically significant. (**C**) Bacterial growth obtained in the blood and peritoneal lavage cultures. Comparisons between groups were performed using Fisher’s exact test.

Microbiological cultures of blood and peritoneal lavage fluid were performed 24 h post-CLP to assess systemic and local bacterial loads. All animals in the CLP group (5/5) had positive blood cultures. In contrast, only one out of five mice in the ATB group and none in the Pg + ATB group had detectable bacteremia. The combination of punicalagin with meropenem led to complete blood culture negativity (0/5), which was statistically significant compared to the CLP group (*p* = 0.0039). The ATB group also showed a significant reduction in bacteremia compared to the CLP group (*p* = 0.0476) (Figure 2A). Positive peritoneal cultures were observed in all CLP mice (5/5), whereas the ATB and Pg + ATB groups had 3/5 and 2/5 positive cultures, respectively. Although both treatment groups showed reductions, the differences were not statistically significant (CLP vs. ATB, *p* = 0.206; CLP vs. Pg + ATB, *p* = 0.083) (Figure 2B). A representation of the bacterial growth obtained in the blood and peritoneal lavage cultures is shown in Figure 2C.

### 2.2. Hematological Changes

There was no significant difference in the hematological parameters of the group treated with the combination of antibiotics and punicalagin compared with the others. However, the CLP group showed an increase in PDW (*p* < 0.05) compared to the Sham group (Table 1).

### 2.3. Effect of Pg ATB Treatment on Lymphoid Organs

**Figure 3:**
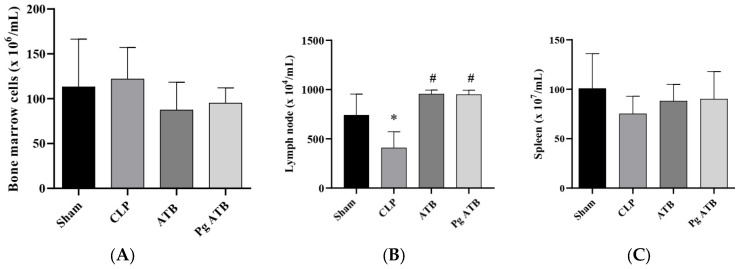
Number of leukocytes in the lymphoid organs of septic mice: (**A**) bone marrow; (**B**) mesenteric lymph nodes; (**C**) spleen. The animals were divided into four groups: Sham, animals that underwent the surgical process but without cecal ligation and without treatment; CLP, animals that had sepsis induced by cecal ligation and perforation without treatment; ATB, animals that had sepsis induced by cecal ligation and perforation and were treated with meropenem; and Pg ATB, animals that had sepsis induced by cecal ligation and perforation and were treated with Pg and ATB. ANOVA statistical tests were used, followed by the Kruskal–Wallis post-test, and significant differences are represented by * in relation to the sham group and # in relation to the CLP group. Data are presented as mean ± SD, with *p* < 0.05 as the significance level.

Pg ATB treatment did not induce changes in the bone marrow (Figure 3A) and spleen (Figure 3C) cell numbers, but this led to a significant increase in the number of mesenteric lymph node cells when compared to CLP (*p* < 0.001) (Figure 3B). Moreover, there was a reduction in the number of cells in the CLP group compared to that in the sham group (*p* < 0.05) (Figure 3B).

### 2.4. Treatment with Pg ATB Influenced the Number of Leukocytes in the Peritoneum

**Figure 4:**
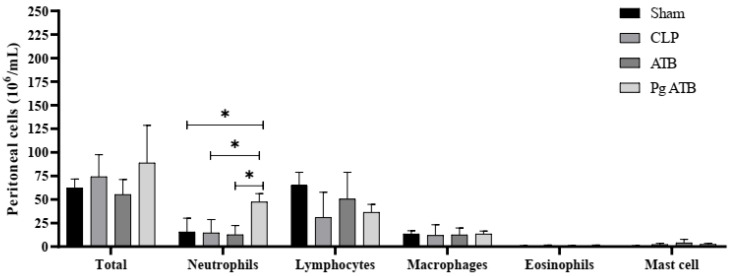
Total and differential numbers of leukocytes in the peritoneum of septic mice. The animals were divided into four groups: Sham, animals that underwent the surgical process, but without cecal ligation and without treatment; CLP, animals that had sepsis induced by cecal ligation and perforation without treatment; ATB, animals that had sepsis induced by cecal ligation and perforation and treatment with meropenem; and Pg ATB, animals that had sepsis induced by cecal ligation and perforation and treatment with Pg and ATB. ANOVA statistical tests were used, followed by the Kruskal–Wallis post-test, and significant differences are represented by *. The data represent the mean ± SD, adopting *p* < 0.05 as significance.

Treatment with Pg ATB significantly increased the number of neutrophils in the peritoneum of mice compared to that in the Sham (*p* < 0.0305) and CLP (*p* < 0.0085) groups for the same cell type (Figure 4).

### 2.5. Effect of Pg ATB Treatment on Liver, Kidney, Intestine, and Lung

**Figure 5:**
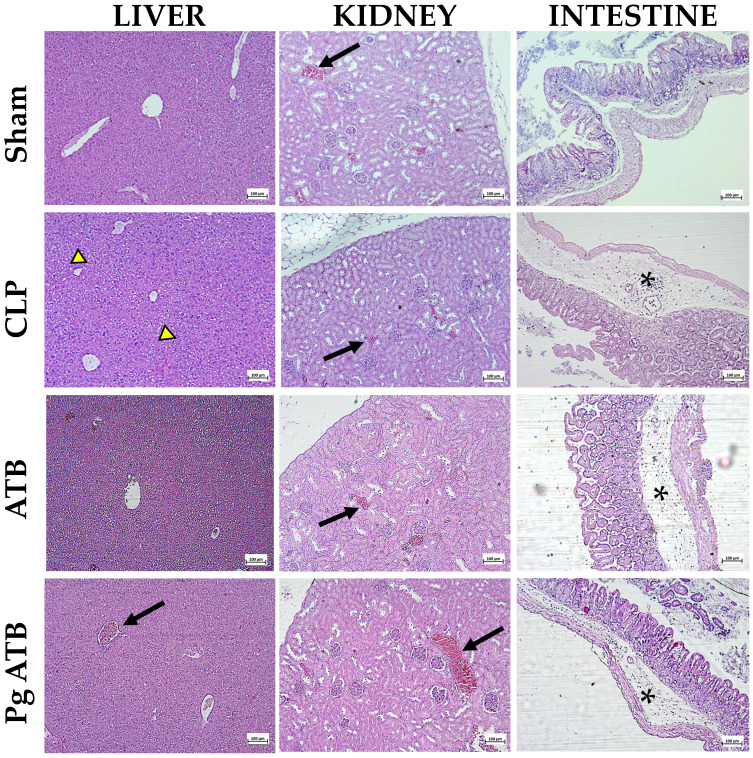
Histological examination of liver, kidney, and intestinal tissue samples from mice subjected to sublethal sepsis. The animals were classified into four groups: Sham, which included animals that underwent surgical procedures but did not receive cecal ligation or treatment; CLP, which included animals that experienced sepsis through cecal ligation and perforation without treatment; ATB, animals that had sepsis induced by cecal ligation and perforation and were treated with meropenem; and Pg ATB, which included animals that developed sepsis through cecal ligation and perforation and received treatment with Pg and ATB. Congestion (black arrow), degeneration (yellow arrow), and inflammatory infiltrate (asterisk) were observed. Hematoxylin and eosin (HE) staining were performed, and images were captured at a resolution of 100 µm.

Histological analysis revealed changes in the livers of untreated septic rats (CLP). Specifically, some hepatic lobules were paler, indicating hydropic degeneration, and there was intense congestion. In contrast, the treated group showed no significant histological changes and only moderate congestion (Figure 5). The kidneys of the CLP group showed slight paleness in the medullary tubules and intense congestion in the tubules. In the ATB-treated group, congestion was identified, while in the Pg + ATB-treated group, although intense congestion was also observed, no significant histological changes were detected (Figure 5). In the intestines of the CLP group animals, there was moderate inflammatory infiltrate in all four layers (mucosa, submucosa, muscularis, and serosa), with a predominance in the submucosa. Some areas showed moderate congestion with hemorrhage, and the collagen fibers in the submucosa were separated, indicating an edematous process. In contrast, the ATB- and Pg ATB-treated groups showed mild inflammatory infiltrate present in all four layers, predominantly in the submucosa, but without hemorrhage (Figure 5).

**Figure 6:**
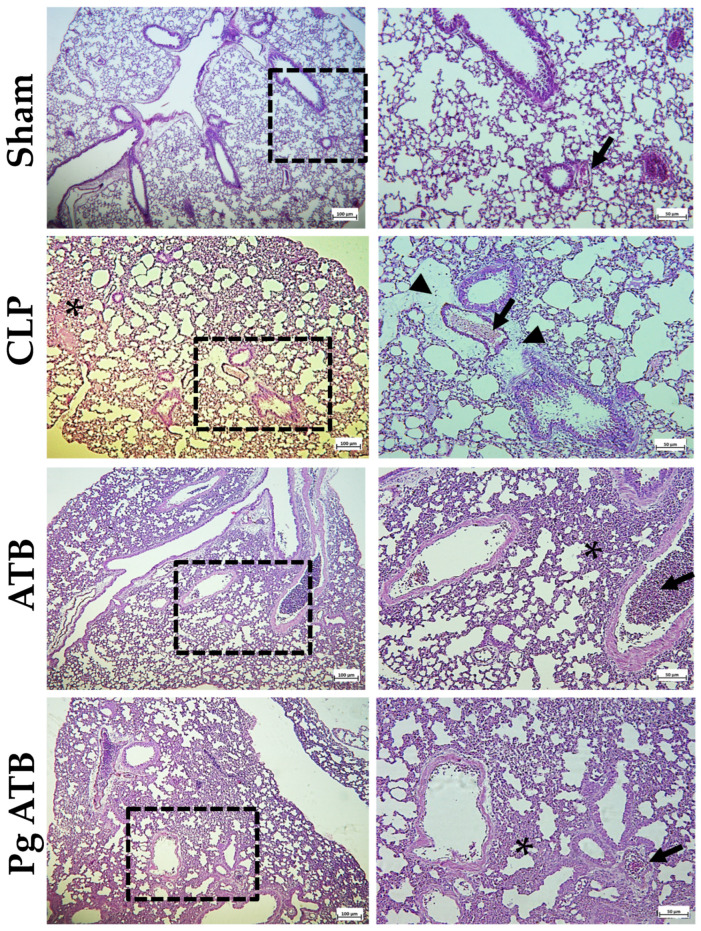
Histological analysis of the lungs of mice subjected to sublethal sepsis. The animals were divided into four groups: Sham, animals that underwent the surgical process but without cecal ligation and without treatment; CLP, animals that had sepsis induced by cecal ligation and perforation, without treatment; ATB, animals that had sepsis induced by cecal ligation and perforation and were treated with meropenem; and Pg ATB, animals that had sepsis induced by cecal ligation and perforation and were treated with Pg and ATB. Presence of congestion (black arrow), inflammatory infiltrate (asterisk), and edema (arrowhead). HE. Scale bars = 100 µm (**left**) and 50 µm (**right**). The images on the right refer to a higher magnification of the hatched area on the left.

In the lungs, the CLP group presented thickening of the interalveolar septa, with greater cellularity, characterizing diffuse and moderate interstitial infiltration, composed mainly of mononuclear cells, in addition to moderate congestion and edema. The groups treated with ATB and Pg ATB did not present signs of edema; however, interstitial infiltration in the interalveolar septa and congestion were also observed (Figure 6).

### 2.6. Cytokine Production

The analysis of cytokine levels, including Interleukin-2 (IL-2), Interleukin-4 (IL-4), Interleukin-6 (IL-6), Interferon-gamma (IFN-γ), Tumor Necrosis Factor-alpha (TNF-α), Interleukin-17 (IL-17), and Interleukin-10 (IL-10), was performed at the systemic level in serum obtained from blood. The results indicated that treatment with Pg ATB led to increased production of the anti-inflammatory cytokine IL-10 at the systemic level (*p* < 0.05) (Table 2).

## 3. Discussion

Sepsis is a life-threatening syndrome characterized by a dysregulated host response to infection, leading to widespread inflammation, microcirculatory dysfunction, tissue hypoxia, and ultimately, multi-organ failure [19,20]. Nitric oxide (NO) is a key mediator of sepsis pathophysiology, exerting both protective and detrimental effects on the host. While basal NO production maintains vascular tone and immune surveillance, excessive or unregulated NO, particularly via inducible nitric oxide synthase (iNOS), can contribute to vasodilation, hypotension, and cellular injury [21]. Given the pivotal role of NO, exploring therapeutic strategies to modulate its levels is essential.

In this context, meropenem, a broad-spectrum carbapenem antibiotic, is frequently used to manage sepsis because of its bactericidal activity. However, evidence suggests that its therapeutic efficacy may be limited when used in monotherapy. For instance, Yeh et al. [22] reported that meropenem treatment alone led to a survival rate of only 37.5% in mice with sepsis. Although meropenem effectively reduces bacterial loads in both feces and peritoneal exudates [23], it lacks immunomodulatory properties. De Araujo et al. [24] observed that animals treated solely with meropenem exhibited severe clinical deterioration within 24 h, persistent neutrophilia, high levels of NETs and myeloperoxidase, and sustained production of inflammatory cytokines. Moreover, meropenem did not reduce serum markers of tissue damage, such as LDH, ALT, urea, or lactate, nor did it elevate IL-10 levels, indicating insufficient systemic inflammation control.

Taken together, these findings highlight a critical limitation: meropenem alone fails to attenuate the excessive inflammatory response and does not prevent multi-organ dysfunction, merely slowing the kinetics of death. This reinforces the need for combinatorial therapies that control infection and modulate the host immune response.

Therefore, this study proposes a novel therapeutic strategy involving the combination of punicalagin (Pg), a polyphenolic compound with antioxidant and immunomodulatory effects, and meropenem. Pg has a unique ellagitannin structure that can interact with microbial membranes and modulate redox-sensitive signaling pathways. We hypothesized that Pg can act as an adjuvant, enhancing the therapeutic impact of meropenem by modulating immune responses, preserving tissue integrity, and promoting bacterial clearance.

In our experimental model, treatment with Pg and meropenem (Pg ATB) significantly modulated the NO levels in septic mice (Figure 1A,B). Specifically, Pg ATB reduced NO levels in the mesenteric lymph nodes, likely via iNOS suppression, while increasing NO production in the peritoneal cavity, potentially enhancing local bacterial clearance [25,26,27,28]. This site-specific regulation of NO may reflect improved control of infection at the primary site while avoiding systemic inflammatory injuries.

In addition to NO modulation, Pg ATB markedly reduced bacterial translocation and peritoneal bacterial growth (Figure 2). The findings of this study provide compelling evidence that punicalagin, when combined with meropenem, exerts a potent synergistic effect in controlling systemic infections in a murine model of sublethal sepsis. The complete absence of bacteremia in the Pg ATB group was biologically significant and statistically robust, especially considering the consistent positivity in the untreated controls. The antimicrobial activity of punicalagin has been previously reported against a broad range of pathogens, including multidrug-resistant strains, through mechanisms such as membrane disruption and metal ion chelation [29,30]. While meropenem acts by inhibiting bacterial cell wall synthesis, punicalagin disrupts membrane integrity, induces acidification, and may interfere with resistance mechanisms [10,31]. This synergy could be further enhanced by Pg’s ability to penetrate biofilms or suppress efflux pumps, which warrants future mechanistic exploration.

Although peritoneal culture results did not reach statistical significance, the numerical trend supports a possible local benefit of this combination therapy. These data suggest that punicalagin could serve as a valuable adjunct to conventional antibiotic therapy in sepsis management, meriting further investigation in larger cohorts and mechanistic studies.

The systemic benefits of Pg ATB were evident in the attenuation of platelet distribution width (PDW), a surrogate of coagulopathy, and preservation of lymphoid architecture, suggesting protection against sepsis-induced coagulopathy and immunosuppression [32,33,34,35,36,37]. Furthermore, an increase in peritoneal neutrophils without systemic leukocytosis indicates effective immune cell recruitment and infection containment [38,39].

Histological analyses revealed reduced tissue injury in multiple organs (Figure 5 and Figure 6). The anti-inflammatory effects of punicalagin, including NF-κB inhibition and cytokine suppression, likely underpin these findings [40,41,42,43]. The observed increase in IL-10 levels supports the notion of balanced immunomodulation, allowing pathogen clearance while limiting collateral damage [44,45].

In summary, our findings demonstrate that punicalagin, when combined with meropenem, offers the dual benefits of enhanced bacterial control and regulation of host immune responses. This therapeutic synergy represents a promising strategy to overcome the limitations of antibiotic monotherapy in sepsis, offering new perspectives for adjunctive therapy in critical-care settings.

## 4. Materials and Methods

### 4.1. Treatment

Pg was obtained from Sigma-Aldrich^®^ (Burlington, MA, USA), stored at 2–8 °C until use, and diluted in sterile Phosphate-Buffered Saline (PBS). For animal treatment, Pg 500 µg/mL [46] was used in a single dose after sepsis associated with meropenem 10 mg/kg [47], 6 h after sepsis induction.

### 4.2. Animals

A total of 15 Swiss male mice (2–3 months, 20–25 g) were obtained from the Central Animal House of Federal University of Maranhão (UFMA) and kept in the animal house of the Laboratory of Immunophysiology in a controlled environment under a 12/12 h light/dark cycle. Water and food were provided ad libitum until the day of sacrifice. The animals were handled according to the Brazilian Society for Laboratory Animal Science (SBCAL) guidelines and the UFMA Research Ethics Committee (protocol number 23115.031277/2019-19) approved the study.

### 4.3. Experimental Design

The animals were divided into four groups. The first group, Sham (surgical procedure without perforation, *n* = 5), received only sterile PBS. The other group underwent CLP (induction of sublethal sepsis by cecal ligation and perforation, *n* = 5) and received 1 mL of sterile 0.9% saline solution subcutaneously. The third group, Pg ATB (septic mice that received Pg associated with the antibiotic, *n* = 5), received 100 µL of Pg (500 µg/mL) associated with meropenem 10 mg/kg subcutaneously 6 h after the procedure [46]. In our previous experience, five animals per group is the minimum number that guarantees statistical significance [48]. The animals in each group were housed in one cage. In the experiment, confounding factors such as the sequence of treatments and location of the animal cages were managed. The experimental units were assigned to the Sham, CLP, and Pg ATB groups using simple randomization. Researchers directly involved in conducting the animal experiments were blinded to the group assignments of the mice. For histopathological analysis, samples from each organ of three randomly selected animals from each group were examined. Conversely, for the other tests, samples from all five animals in each group were analyzed.

Animals were anesthetized with 2% xylazine (Rompum^®^, 20 mg/kg, Bayer Animal Health, Leverkusen, Germany) and 5% ketamine (Vetanarcol^®^, 25 mg/kg, Laboratorios Konig S. A., Avelleda, Argentina) intraperitoneally. A laparotomy was performed, and the cecum was mobilized and ligated below the cecal valve and punctured five times with an 18-gauge needle to induce sublethal sepsis [49], with adaptations to the original protocol. The cecum was replaced in the peritoneal cavity, and the abdomen was closed in two layers. Sterile saline (1 mL) was subcutaneously administered for resuscitation. Twenty-four hours after the procedure, all animals were sacrificed with an overdose of anesthetic (150 mg/kg ketamine hydrochloride and 120 mg/kg xylazine hydrochloride) to carry out the tests.

### 4.4. Total and Differential Cell Count

To count the total number of cells, cell suspensions were stained with crystal violet (0.05%) and 30% acetic acid in a 9:1 ratio and counted in a Neubauer chamber under an optical microscope at 400× magnification under common light. The differential counts of these cells were determined using a cytospin system (800 rpm/3 min), fixed, and stained using the Instant-Prov kit (Newprov, Pinhais, Brazil). The percentage of cell subpopulations was calculated based on a 100-cell count and transformed into an absolute number based on the total count.

### 4.5. Hematological Parameter Assessment

After anesthesia, blood samples were collected from the retro-orbital plexus and transferred to conical plastic tubes containing EDTA. Blood smears were prepared with 10 μL of blood and stained using the Instant-Prov kit (Newprov, Pinhais, Brazil). The slides were examined under an ordinary light microscope at 100× magnification. The percentage of cell subpopulations (WBC differential count) was calculated based on a 100-cell count. Red blood cell count (RBC), mean corpuscular volume (MCV), red cell distribution width measured as coefficient of variation (RDW-CV), red cell distribution width measured as standard deviation (RDW-SD), hematocrit (HCT), platelet count (PLT), mean platelet volume (MPV), platelet distribution width (PDW), plateletcrit (PCT), and platelet–large cell ratio (P-CSF) were estimated using the Hemogram^®^ Hemacounter 60 (São Paulo, Brazil).

### 4.6. Histopathological Analyzes

The lungs, kidneys, liver, and intestines were collected and fixed in 10% formalin, embedded in paraffin, cut into microtome sections of 5 μm thickness, and stained with hematoxylin and eosin for histology. The slides were examined under a light microscope at 20×, 40×, and 100×. Histopathological analysis was used to assess edema, vascular congestion, hemorrhage, and inflammatory infiltrates. Histological assessments were performed randomly and blindly.

### 4.7. Determination of Bacteria in Biological Samples

To assess the presence of bacteria in the blood and peritoneal lavage, 10 μL of the exudate was collected, and serial decimal dilutions were performed in 96-well plates containing 90 μL of PBS in each well. Then, two aliquots of 10 μL of these dilutions of each sample were seeded on Brain Heart Infusion (BHI) agar plates using the microdrop technique, and the experiments were performed in quadruplicate [50]. The plates were incubated at 37 °C for 24 h to allow bacterial colony growth.

### 4.8. Spleen, Lymph Node, and Bone Marrow Cell Count

After euthanasia, the femurs, spleens, and mesenteric lymph nodes were removed from the mice. The femur was perfused with 1 mL PBS to isolate the bone marrow cells. The spleen was removed, triturated with 5 mL of PBS, and passed through a silk sieve. The mesenteric lymph nodes were removed and minced in 1 mL Roswell Park Memorial Institute (RPMI) medium supplemented with L-glutamine and 10% fetal bovine serum. Nine cell suspension volumes were added to one volume of 0.05% crystal violet dissolved in 30% acetic acid, and the cells were counted using a Neubauer chamber under standard light microscopy at 40× magnification.

### 4.9. Determination of Serum Cytokines and Peritoneal Lavage

Cytometry was performed using the BD™ Cytometric Bead Array (CBA) kit (Mouse Th1/Th2/Th17, catalog number 560485, San Jose, CA, USA) for quantification of IL-2, IL-4, IL-6, IFN-γ, TNF-α, IL-17A, and IL-10. A mouse inflammatory cytokine kit was purchased from Becton Dickinson Biosciences (San Jose, CA, USA). Serum or peritoneal lavage samples, obtained 12 h after CLP, were centrifuged at 1.500 rpm for 10 min at room temperature to precipitate debris with the detection reagent from the kit. The samples were incubated for 2 h at room temperature and protected from light. Subsequently, the samples were suspended in 500 mL of washing buffer in each test tube and centrifuged at 200× *g* for 5 min. Samples were suspended in 150 mL of wash buffer in each test tube and analyzed using a FACSCalibur flow cytometer (Becton Dickinson). The cytometer was calibrated using three samples of setup beads previously incubated with FITC or PE or without any revealing reagent according to the manufacturer’s instructions. After reading the standards and samples, the data analysis was performed using FCAP Array™ Software version 3.0 (BD Biosciences, San Jose, CA, USA). The results are expressed in pg/mL for each cytokine.

### 4.10. Determination of Nitric Oxide Production

To measure NO, peritoneal lavage and mesenteric lymph node cells were cultured in 100 µL of complete RPMI 1640 medium supplemented with 10 mM HEPES, 11 mM sodium bicarbonate, 100 U/mL penicillin, 100 µg/mL streptomycin, 2 mM L-glutamine, 23 mM L-asparagine, 1 mM folic acid, 0.1 mM pyruvic acid, and 5% fetal calf serum (FCS) for 48 h at 37 °C in a humid atmosphere containing 5% CO_2_ and 95% air. After incubation, 50 μL of the supernatant was collected and incubated with an equal volume of Griess reagent (1% sulfanilamide/0.1% naphthalene diamine dihydrochloride/2.5% H_3_PO_4_) for 10 min at room temperature to quantify the accumulation of nitrite. The absorbance was measured at 550 nm. Absorbance was converted to μM NO by comparison with a standard curve obtained using known concentrations (5–60 μM) of sodium nitrite diluted in RPMI medium [51].

### 4.11. Statistical Analysis

The results are expressed as mean ± standard deviation. Statistical analysis was performed using the Kolmogorov–Smirnov and Shapiro–Wilk normality tests, followed by *t*-test or analysis of variance (ANOVA), Tukey’s post hoc tests, and Dunn’s post hoc test using the GraphPad Prism software, version 8.0. Fisher’s exact test was employed to analyze the microbiological data across the groups. Differences were considered statistically significant at *p* < 0.05. The tests were performed in quadruplicate.

## 5. Conclusions

This study aimed to investigate the efficacy of punicalagin (Pg), a polyphenolic compound with known antimicrobial and anti-inflammatory properties, in combination with meropenem, for the treatment of experimental sepsis induced by cecal ligation and puncture (CLP) in mice. Our findings demonstrated that the Pg–meropenem combination significantly reduced bacterial burden and translocation, particularly in the peritoneum, and modulated NO production in a tissue-specific manner. The treatment also preserved immune organ cellularity, attenuated systemic inflammatory responses, and reduced histopathological damage in key organs, including the liver, kidney, intestine, and lung. Furthermore, it enhanced IL-10 production without impairing neutrophil function, supporting its dual role in promoting pathogen clearance and controlling inflammation.

These results underscore the potential of punicalagin as an adjunctive agent to enhance antibiotic efficacy and immunomodulation during sepsis management. By targeting both microbial and host response pathways, Pg offers a complementary strategy to conventional therapies, particularly in the context of rising antimicrobial resistance and the limitations of current monotherapies.

Nevertheless, the present study was conducted in a murine model of sublethal sepsis and may not fully capture the heterogeneity and complexities of human sepsis. Therefore, future research should focus on (i) elucidating the molecular mechanisms underlying the observed effects; (ii) evaluating efficacy against clinically relevant, multidrug-resistant sepsis pathogens, such as *Klebsiella pneumoniae*, *Escherichia coli*, and *Pseudomonas aeruginosa*; (iii) determining the optimal dosing regimens and therapeutic windows for Pg when used in combination with different antibiotic classes.

Ultimately, clinical trials will be necessary to validate these preclinical findings, assess the pharmacokinetics and safety in humans, and determine the feasibility of integrating punicalagin into standard sepsis treatment protocols. If confirmed, Pg could be a promising component of next-generation adjunctive therapies for sepsis.

## Figures and Tables

**Table 1:** Hematological parameters in septic mice.

Hematological Parameters #	Sham	CLP	ATB	Pg ATB
	Mean ± SD	Mean ± SD	Mean ± SD	Mean ± SD
RBC (×10^6^/µL)	1.44 ± 2.33	5.38 ± 1.16 *	3.22 ± 2.03	4.44 ± 1.23 *
MCV (fL)	41.95 ± 1.47	41.80 ± 0.48	43.76 ± 5.21	41.32 ± 0.73
RDW-CV (%)	14.55 ± 1.29	16.26 ± 1.80	13.44 ± 2.89	16.10 ± 0.63 *
RDW-SD (fL)	30.53 ± 3.19	33.98 ± 3.68	28.76 ± 3.97	33.28 ± 1.30 †
HCT (%)	6.23 ± 10.19	22.56 ± 5.12 *	13.36 ± 8.31	18.32 ± 4.98
PLT (×10^3^/µL)	297.25 ± 315.46	528.40 ± 152.53	286.6 ± 141.7	489.00 ± 65.51 †
MPV (fL)	5.68 ± 0.46	6.02 ± 0.24	5.84 ± 0.31	5.72 ± 0.24
PDW (fL)	5.08 ± 1.86	7.54 ± 0.72 *	7.38 ± 0.67 *	6.72 ± 0.62
PCT (%)	0.17 ± 0.19	0.32 ± 0.09	0.17 ± 0.08	0.28 ± 0.04
P-LCR (%)	7.57 ± 4.57	6.00 ± 1.84	4.64 ± 0.41	4.04 ± 1.41

# RBC, red blood cell count; MCV, mean corpuscular volume; RDW-CV, red cell distribution width measured as coefficient of variation; RDW-SD, red cell distribution width measured as standard deviation; HCT, hematocrit; PLT, platelet; MPV, mean platelet volume; PDW, platelet distribution width; PCT, plateletcrit; P-LCR, platelet–large cell ratio. *t*-test and ANOVA statistical tests were used, followed by Tukey’s post-test, and significant differences are represented by * in comparison with the sham group or † in comparison with the ATB group. Data are presented as mean ± SD, with *p* < 0.05 as significance.

**Table 2:** Serum cytokine content in septic mice.

Cytokines †	Sham	CLP	ATB	Pg ATB
	Mean ± SD	Mean ± SD	Mean ± SD	Mean ± SD
IL-2	1.90 ± 3.80	0.00 ± 0.00	1.64 ± 3.28	0.00 ± 0.00
IL-4	1.02 ± 1.91	0.00 ± 0.00	0.26 ± 0.41	0.00 ± 0.00
IL-6	18.55 ± 13.50	269.7 ± 442.8	4710 ± 4920 *	2423 ± 4913
IFN-γ	0.57 ± 1.10	5.21 ± 9.17	128.5 ± 159.7 *	3.10 ± 3.21
TNF-α	10.69 ± 8.14	39.83 ± 49.48	834.4 ± 843.4 *	116.4 ± 142.3
IL-17	0.31 ± 0.63	0.56 ± 0.80	3.88 ± 3.25	0.77 ± 1.72
IL-10	2.92 ± 5.41	14.76 ± 24.21	1710 ± 1933	39.7 ± 15.9 *,#

† Interleukin-2 (IL-2), Interleukin-4 (IL-4), Interleukin-6 (IL-6), Interferon-gamma (IFN-γ), Tumor Necrosis Factor-alpha (TNF-α), Interleukin-17 (IL-17), and Interleukin-10 (IL-10). The Kruskal–Wallis statistical test was used followed by the Dunn’s post-test or *t*-test and significant differences are represented by * in relation to the Sham group; # in relation to the CLP group. Data represent the mean ± SD adopting *p* < 0.05 as significance.

## Data Availability

The original contributions presented in this study are included in the article. Further inquiries can be directed to the corresponding author.

## Abbreviations

The following abbreviations are used in this manuscript:

ATB	Antibiotic
BHI	Brain Heart Infusion
CBA	Cytometric Bead Array
CFU	Colony-Forming Units
CLP	Cecal Ligation and Puncture
COX-2	Cyclooxygenase-2
FCS	Fetal Calf Serum
HCT	Hematocrit
HE	Hematoxylin and Eosin
HSV-1	Herpes Simplex Virus 1
ICU	Intensive Care Unit
IFN-γ	Interferon-Gamma
iNOS	Inducible Nitric Oxide Synthetase
IL	Interleukin
LPS	Lipopolysaccharid
MCV	Mean Corpuscular Volume
MPV	Mean Platelet Volume
MRSA	Oxacillin in Methicillin-Resistant *Staphylococcus aureus*
NF-κB	Nuclear Factor Kappa B
NK	Natural Killer
NO	Nitric Oxide
PBS	Phosphate-Buffered Saline
PCT	Plateletcrit
PDW	Platelet Distribution Width
P-LCR	Platelet–Large Cell Ratio
PLT	Platelet
Pg	Punicalagin
RBC	Red Blood Cell Count
RDW-CV	Red Cell Distribution Width Measured as Coefficient of Variation
RDW-SD	Red Cell Distribution Width Measured as Standard Deviation
ROS	Reactive Oxygen Species
RSV	Respiratory Syncytial Virus
SARS-CoV-2	Severe Acute Respiratory Syndrome Coronavirus 2
SBCAL	Brazilian Society for Laboratory Animal Science
SD	Standard Deviation
RPMI	Roswell Park Memorial Institute
TNF-α	Tumor Necrosis Factor
